# Magnetoencephalographic source localization and reconstruction via deep learning

**DOI:** 10.3389/fnins.2025.1578473

**Published:** 2025-07-21

**Authors:** Stefano Franceschini, Michele Ambrosanio, Maria Maddalena Autorino, Sohail Maqsood, Fabio Baselice

**Affiliations:** ^1^Department of Engineering, University of Naples Parthenope, Naples, Italy; ^2^Department of Economics, Law, Cybersecurity and Sports Sciences (DiSEGIM), University of Naples Parthenope, Naples, Italy

**Keywords:** beamforming, brain signal estimation, brain source reconstruction, neural networks, magnetoencephalography

## Abstract

Within this manuscript a deep learning algorithm designed to achieve both spatial and temporal source reconstruction based on signals captured by MEG devices is introduced. Brain signal estimation at source level is a significant challenge in magnetoencephalographic (MEG) data processing. Traditional algorithms offer excellent temporal resolution but are limited in spatial resolution due to the inherent ill-posed nature of the problem. Nevertheless, many applications require precise localization of pathological tissues to provide reliable information for clinicians. In this context, deep learning solutions emerge as promising candidates for high resolution signals estimations. The proposed approach, termed “Deep-MEG,” employs a hybrid neural network architecture capable of extracting both temporal and spatial information from signals captured by MEG sensors. The algorithm is capable to handling the entire brain and, therefore, is not limited to cortical sources imaging. To validate its efficacy, the Authors conducted simulations involving multiple active sources using a realistic forward model, and subsequently compared the results with those obtained using various state-of-the-art reconstruction algorithms. Finally Deep-MEG has been tested also with real MEG data.

## 1 Introduction

In the framework of brain functional analysis, Magnetoencephalography (MEG) stands out as one of the main state-of-the-art non-invasive methods for gathering information about brain processing. This acquisition system involves recording magnetic fields using superconducting quantum interference device (SQUID) sensors positioned within a helmet surrounding the patient's head (Cohen, 1972; Kleiner et al., 2004). These magnetic field variations are due to the electrical activity of groups of neurons (i.e., the brain areas), which can be modeled as current dipoles (Mosher et al., 1992). Compared to functional Magnetic Resonance Imaging (fMRI), the MEG system shows an excellent time resolution, while with respect to Electroencephalography (EEG) it is characterized by better spatial resolution given that the layers surrounding the brain do not significantly distort the magnetic field induced by the neuronal activity (Rucco et al., 2020).

After the acquisition, the MEG data is commonly said to be in the signal space. On the opposite, the data in the source space is defined as the signals produced by the brain regions, i.e., the neuronal activity that produced the recorded magnetic fields. Generally, a linear relation is assumed to be between the signals in the two spaces (sources and measurements), described by the so-called leadfiled matrix, which depends on the configuration of sensors as well as the geometric and electric attributes of individual brain anatomy.

One of the first steps in the MEG processing chain, after the denoising and artifact removing, is the solution of the inverse problem, that means the estimation of the brain current sources from the MEG recordings based on the known Leadfield matrix. Unfortunately, this is not a trivial task due to the ill-posedness of the mathematical problem which could lead to a completely erroneous estimation. During the past decades, several strategies for the source reconstruction problem have been proposed in literature. One of them proposes to select the solution that minimizes the L^2^-norm of sources, and called minimum norm estimation (MNE) (Fuchs et al., 1999; Hincapié et al., 2016). Among its derivatives, we cite the low-resolution brain electromagnetic tomography (LORETA) (Pascual-Marqui, 2007; Jun et al., 2019), which adds an a-priori information in order to regularize the solution.

Another family of source reconstruction algorithms are the so-called beamformers. In brief, this methods scan a set of predefined putative source locations by means of spatial filters to pass signals selectively from desired locations while suppressing activity from other brain regions (Westner et al., 2022). Among all, we recall the synthetic aperture magnetometry (SAM) (Robinson, 1999) and the linearly constrained minimum variance (LCMV) (Van Veen et al., 1997). The main issue of both these approaches relies on the poor spatial resolution. In order to mitigate such problem, algorithms incorporating a variety of a-priori information and regularization strategies have been proposed, leading to adaptive beamformers approaches. Moreover, another limitation of LCMV beamformer is its inability to accurately reconstruct correlated sources. Some of these algorithms exploit noise covariance matrices to achieve adaptation (Hossein et al., 2018; Nunes et al., 2020; Moiseev et al., 2022).

Another family of source reconstruction algorithms is the multiple signal classification (MUSIC), which replaces the multiple-dipole directed search with a single-dipole scanning procedure confined to a three-dimensional head or source volume (Mosher and Leahy, 1998; Ermer et al., 2000).

In recent years, the scientific community has shown a considerable interest into deep learning (DL), a family of algorithms initially developed for computer vision tasks (LeCun et al., 2015; Ferraioli et al., 2019). This approach has gained traction in various clinical contexts (Litjens et al., 2017; Zemouri et al., 2019). Among all, we cite the neural networks in MRI signal analysis (Pereira et al., 2016; Autorino et al., 2024), in PET-CT imaging Given these advantages, DL methods have been widely adopted in brain source localization and reconstruction. For example, Hecker et al. (2021) proposed a convolutional neural network for EEG source imaging, while Pantazis and Adler (2021) investigated neural network-based source reconstruction for both instantaneous and time series MEG signals. An edge sparse basis network is employed for EEG source localization and is presented in Wei et al. (1 01). Moreover, a DL solution for localizing epileptogenic zones based on MEG interictal spikes is introduced by Sun et al. (2023), and Liang et al. (2023) focused on brain source imaging using sparse Bayesian learning within a DL framework. Furthermore, DL has been applied for both localization and reconstruction tasks, as demonstrated in Yu et al. (2024), which presents a DL approach for reconstructing EEG data in the context of epilepsy. Furthermore, for the best of Authors' knowledge, Deep-MEG is the first deep-learning-based method capable to deal with the whole parenchyma brain and, therefore, with deep sources.

Within this manuscript, a novel DL framework for brain source localization and reconstruction, termed “Deep-MEG”, is presented. The proposed solution is a hybrid neural network consisting of a cascade of convolutional layers followed by fully-connected (FC) layers. This architecture allows the network to incorporate both temporal and spatial information. Deep-MEG adopts an end-to-end approach, directly reconstructing sources from signals collected at MEG sensor locations. Unlike the method proposed in Yu et al. (2024), which operates on EEG signals and is limited to retrieving signals from cortical dipoles, our approach operates on MEG signals and can estimate signals from dipoles located throughout the entire brain volume. Furthermore, while Yu et al. (2024) focuses on scenarios where an active dipole generates spike signals indicative of epilepsy, our simulations encompass a broader range of signals (further details are provided in Section 3).

It is noteworthy that the proposed solution operates with only the Leadfield matrix and a short time window of MEG data, eliminating the need for covariance or spectral density matrix estimation. This design choice makes the approach robust against potential estimation errors. Deep-MEG has undergone testing across various simulated scenarios featuring multiple punctual and extended sources. Comparative evaluations against several state-of-the-art source reconstruction algorithms consistently demonstrate its superiority in terms of both spatial and temporal resolution. Furthermore, the proposed approach has been tested with real MEG data from the open source database “OpenNEURO” (Henson et al., 2011; Wakeman and Henson, 2015). More details on the processing and adopted data are reported in Section 4.6.

The manuscript is organized as it follows: Section 2 provides a mathematical description of the brain imaging problem, while Section 3 delves into the proposed solution in greater detail. Section 4 presents and discusses the results derived from validation tests in both numerical and real scenarios, considering single focal sources and extended areas of the active brain. Within Section 5 a discussion about the main advantages and main limitations of the proposed solution. Finally, Section 6 concludes the paper with some closing remarks.

## 2 Mathematical formulation and problem statement

MEG is a non-invasive and safe technique used to measure the magnetic fields generated by the brain, providing real-time insights into neural activity. These electromagnetic signals originate from the electrical currents flowing through the apical dendrites of pyramidal neurons in the cerebral cortex. At a sufficient distance, the simultaneous activation of thousands of cortical neurons can be represented as an equivalent current dipole. This dipole serves as the fundamental unit for modeling neural activation, with the entire brain conceptualized as a collection of hundreds or thousands of such dipoles, depending on spatial resolution. By solving Maxwell's equations in a quasi-static regime (which involves frequencies lower than 100 Hz), the quasi-static current density **J**(**r**′) at position **r**′ can be related to the induced magnetic field **B**(**r**) at position **r** through the Biot-Savart law (Baillet et al., 2001):


(1) $$\begin{array}{l} \text{B}\left(\text{r}\right)=\frac{\mu_{0}}{4\pi }\int_{V}\text{J}\left({\text{r}}^{\prime }\right)\times \frac{\text{r}-{\text{r}}^{\prime }}{||\text{r}-{\text{r}}^{\prime }{||}^{3}}d{\text{r}}^{\prime }, \end{array}$$

where μ_0_ represents the free space magnetic permeability equal to 4π·10^−7^ H/m, the symbol × refers to the cross product between vectors, ||(·)|| refers to the L^2^-norm, and the integral in Equation 1 extends over the volume V encompassing the currents. It is noteworthy to observe that bold quantities refer to vectors, matrices and discretized quantities depending on the context.

Two distinct current contributions can be discerned: a primary current density arising from neuronal activity and a volume one associated with the effects of magnetic fields within the surrounding tissue volume:


(2) $$\begin{array}{l} \text{J}\left(\text{r}\right)={\text{J}}^{P}\left(\text{r}\right)+{\text{J}}^{V}\left(\text{r}\right)={\text{J}}^{P}\left(\text{r}\right)+\sigma \left(\text{r}\right)\text{E}\left(\text{r}\right)\\ \;={\text{J}}^{P}\left(\text{r}\right)-\sigma \left(\text{r}\right)\nabla V\left(\text{r}\right), \end{array}$$

where σ(**r**) is the electrical conductivity profile of the head tissues (assumed to be isotropic) and the electric field **E**(**r**) is the negative gradient of the electric potential *V*(**r**).

Assuming that the head can be modeled as a collection of contiguous regions with isotropic conductivity σ_*i*_, where *i* = {1, 2, 3}, representing different tissues such as brain, skull, and scalp, the Biot-Savart equation can be reformulated as follows:


(3) $$B(r)=B_{0}(r)+\frac{\mu_{0}}{4\pi }\sum_{ij}\left(\sigma_{i}-\sigma_{j}\right)\int_{S_{ij}}V(r^{\prime })\frac{r-r^{\prime }}{||r-r^{\prime }{||}^{3}}\times dr^{\prime },$$

where **B**_0_(**r**) represents the induced magnetic field due to the primary current and *S*_*ij*_ represent the boundary surface between two isotropic region. The second contribution, related to the volume current, is the sum of the surface integrals over every isotropic head region (brain, skull and scalp). By following a similar procedure, it is possible to derive a corresponding equation for the potential *V*(**r**):


(4) $$\begin{array}{l} \left(\sigma_{i}+\sigma_{j}\right)V\left(\text{r}\right)=2\sigma_{0}V_{0}\left(\text{r}\right)\\ -\frac{1}{2\pi }\underset{ij}{\sum }\left(\sigma_{i}-\sigma_{j}\right)\int_{S_{ij}}V\left({\text{r}}^{\prime }\right)\frac{\text{r}-{\text{r}}^{\prime }}{||\text{r}-{\text{r}}^{\prime }{||}^{3}}\cdot d{\text{r}}^{\prime }, \end{array}$$

where · refers to the dot product, *V*_0_(*r*) is the potential related to the primary current and σ_0_ is the unitary conductivity needed for coherent dimensional analysis. Equations 3, 4 can be utilized to address the forward problem. By specifying the value of the primary current **J**^*P*^(**r**), it is possible to calculate the primary potential and magnetic field:


(5) $$\begin{array}{l} V_{0}\left(\text{r}\right)=\frac{1}{4\pi \sigma_{0}}\int {\text{J}}^{P}\left({\text{r}}^{\prime }\right)\cdot \frac{\text{r}-{\text{r}}^{\prime }}{||\text{r}-{\text{r}}^{\prime }{||}^{3}}d{\text{r}}^{\prime }, \end{array}$$

(6) $$\begin{array}{l} {\text{B}}_{0}\left(r\right)=\frac{\mu_{0}}{4\pi }\int {\text{J}}^{P}\left({\text{r}}^{\prime }\right)\times \frac{\text{r}-{\text{r}}^{\prime }}{||\text{r}-{\text{r}}^{\prime }{||}^{3}}d{\text{r}}^{\prime }. \end{array}$$

The primary potential *V*_0_(**r**) evaluated in Equation 5 is subsequently employed to address Equation 4 for determining the potentials across all surfaces, thereby resolving the forward problem. These surface potentials *V*(**r**) and the primary magnetic field **B**_0_(**r**) of Equation 6 are then utilized to tackle Equation 3 for the external induced magnetic field. However, it is important to note that the solution of Equation 4 possesses analytical solutions only for specific shapes and must otherwise be solved numerically. A common approximation involves modeling the head shape as a multi-shell spherical structure. Under this assumption, if a dipole with moment **q** is positioned at **r**_*q*_, and the MEG system measures only the radial component of the magnetic field **B**_*r*_(**r**) at a certain point **r**, the volumetric component of the field becomes negligible, resulting in only the primary term **B**_0_(**r**). Taking the radial component of this field for the current dipole simplifies to the remarkably straightforward expression:


(7) $$B_{r}(r)=\frac{r}{r}\cdot B(r)=\frac{r}{r}\cdot B_{0}(r)=\frac{\mu_{0}}{4\pi }\frac{r\times r_{q}}{r||r-r_{q}{||}^{3}}\cdot q,$$

in which *r* is the magnitude of the vector **r**, and **r**_*q*_ identifies the dipole position. Equation 7 is linear with respect to **q** and highly non-linear with respect to its location **r**_*q*_. Considering separately the magnitude of a dipole *Q* = ||**q**|| from its orientation **Θ** = **q**/||**q**||, the magnetic field generated by the dipole can be written as:


(8) $$\begin{array}{l} M\left(\text{r}\right)=L\left(\text{r},{\text{r}}_{q},\text{Θ}\right)Q, \end{array}$$

and in case of multiple dipoles:


(9) $$\begin{array}{l} M\left(\text{r}\right)=\underset{i}{\sum }L\left(\text{r},{\text{r}}_{qi},{\text{Θ}}_{i}\right)Q_{i}. \end{array}$$

In the case of a complete MEG system equipped with numerous sensors, Equation 9 becomes Malmivuo and Plonsey (1995):


(10) $$\begin{array}{l} \left[\begin{array}{c} M\left({\text{r}}_{1}\right)\\ \vdots \\ M\left({\text{r}}_{S}\right) \end{array}\right]=\left[\begin{array}{ccc} L\left({\text{r}}_{1},{\text{r}}_{q1},{\text{Θ}}_{1}\right) & \cdots & L\left({\text{r}}_{1},{\text{r}}_{qD},{\text{Θ}}_{D}\right)\\ \vdots & \ddots & \vdots \\ L\left({\text{r}}_{S},{\text{r}}_{q1},{\text{Θ}}_{1}\right) & \cdots & L\left({\text{r}}_{S},{\text{r}}_{qD},{\text{Θ}}_{D}\right) \end{array}\right]\left[\begin{array}{c} Q_{1}\\ \vdots \\ Q_{D} \end{array}\right]. \end{array}$$

Equation 10 can be written in its compact (matrix) form as:


(11) $$\begin{array}{l} \text{M}=\text{L}\text{Q}+\text{N}, \end{array}$$

in which **N** represents some additive noise. In Equation 11, the brain volume is discretized into *D* regions, with a MEG helmet comprising *S* magnetometers, and each acquisition is assumed to be composed of *T* time samples. The matrix **M**∈ℝ^*S* × *T*^ represents the acquisition matrix containing all the signals recorded by the sensors. **Q**∈ℝ^*D* × *T*^ denotes the matrix of all the current dipoles generated in the brain, while **L**∈ℝ^*S* × *D*^ serves as the forward matrix linking brain and sensor signals, commonly referred to as the *Leadfield* matrix. Additionally, **N**∈ℝ^*S* × *T*^ represents the measurement noise, often modeled as white Gaussian noise. Notably, the Leadfield matrix inherently incorporates information about the acquisition system, such as the head geometry, orientation of dipoles, distance between sources and sensors, and the presence of skull and skin.

**Figure 1:**
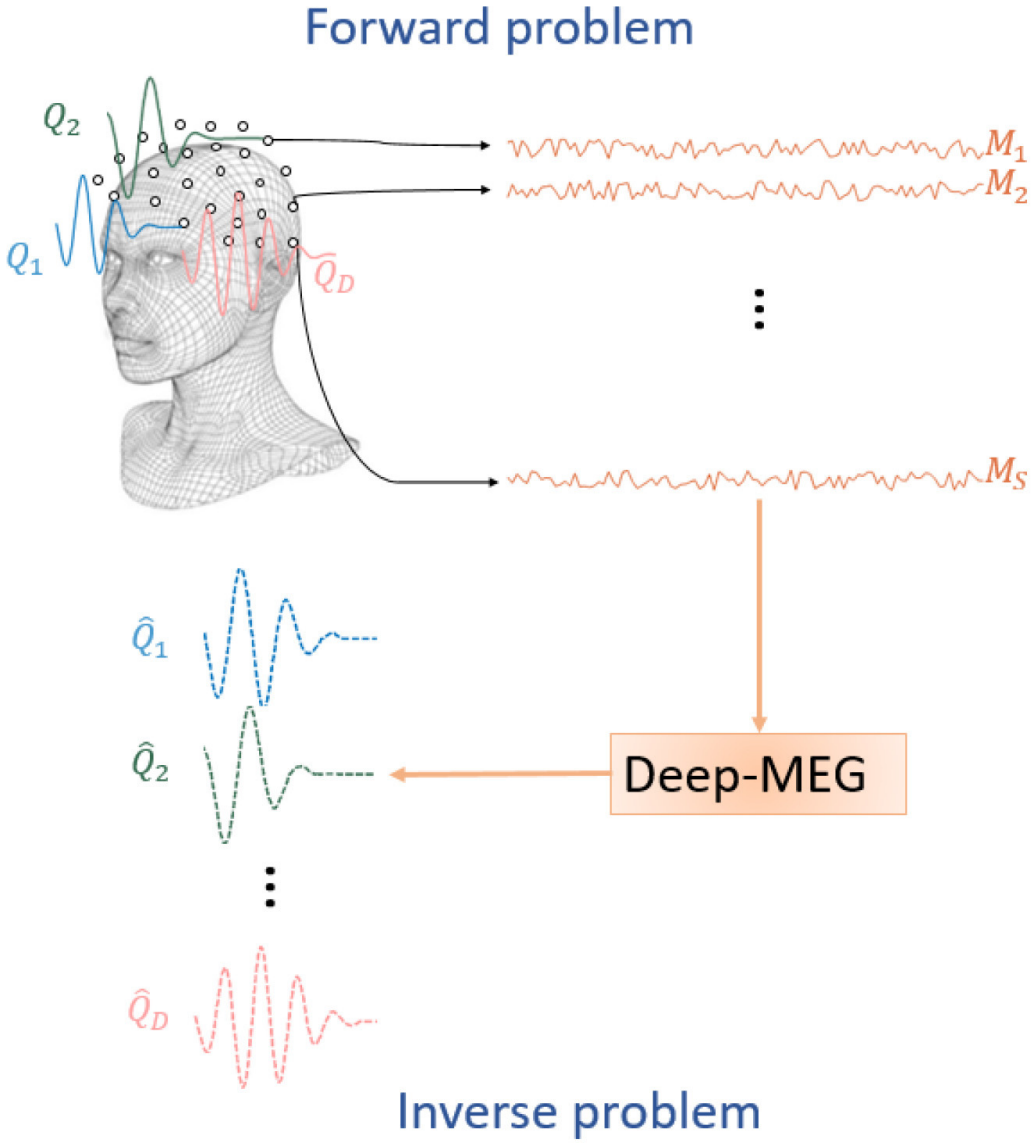
Sketch of MEG signal acquisition and reconstruction. The brain electrical activity [*Q*_1_, *Q*_2_, ..., *Q*_*D*_] is measured by the magnetometers located around the scalp and producing the magnetic signals [*M*_1_, *M*_2_, ..., *M*_*S*_]. Such signals are the input of the proposed Deep-MEG approach which provides as output an estimation of the brain electrical activity [${\hat{Q}}_{1},{\hat{Q}}_{2},...,{\hat{Q}}_{D}$].

In the context of the model described by Equation 11, the brain source reconstruction problem entails deriving an estimate of the matrix **Q** based on the acquired data **M**. Figure 1 provides a schematic representation of the forward and inverse MEG problems. In practical scenarios, the number of dipoles *D* significantly exceeds the number of sensors *S*, rendering the problem ill-posed and lacking a unique solution. Consequently, it is paramount to adopt approximations and a-priori information to perform the inversion properly. In this framework, various techniques have been adopted in the scientific literature, including singular value decomposition (SVD), Markov and Bayesian models, and adaptive beamformers (Jonmohamadi et al., 2014; Senaratne and Tellambura, 2010; Woolrich et al., 2011; Cai et al., 2023).

In the proposed work, a solution to the aforementioned problem is achieved by means of a deep learning hybrid model, comprising a first block of convolutional layers for the extraction of temporal information, and a second block made of FC layers for the spatial information retrieval. Further details regarding the proposed Deep-MEG architecture are presented in the following Section.

## 3 Deep-MEG

### 3.1 Architecture details

**Figure 2:**
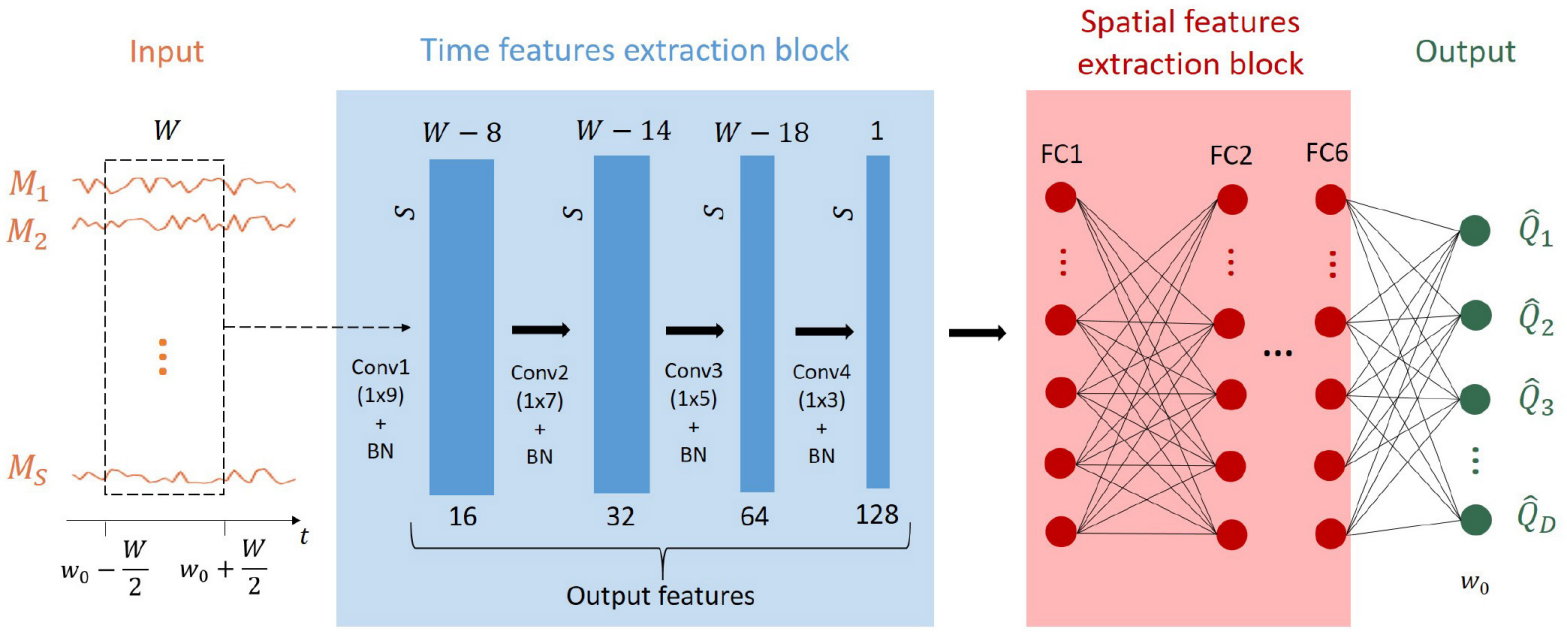
Sketch of the proposed Deep-MEG brain source localization and signal reconstruction approach. The model takes as input a window of *W* samples (centered in the sample *w*_0_) of the *S* MEG signals. It is composed of a cascade of CNN layers which extract temporal features, followed by some fully-connected layers extracting spatial information. The output of the approach are the retrieved brain signals for all the *D* brain areas at the window center.

Deep-MEG is an end-to-end artificial neural network designed for brain source localization and brain signal reconstruction exploiting MEG data. A block diagram of the proposed architecture is shown in Figure 2.

A time window extracts *W* samples from each of the *S* MEG acquired signals. This results in an *S* × *W* matrix, which serves as the input for the first block of the network. This section of the algorithm consists of 4 convolutional layers responsible for extracting time features. At each layer, the temporal dimension of the data is reduced while the number of features progressively increases. A batch normalization layer is present between each convolutional layer and the following one. The final convolutional layer produces a matrix composed of *S* vectors and 128 features. This matrix is then flattened and fed into a sequence of 6 FC layers, each consisting of 500 nodes. Each FC layer is followed by a Rectified Linear Unit (ReLU) activation function. The last layer is a FC layer composed of *D* nodes. The output of the proposed architecture is a vector of *D* samples representing the amplitude of the brain signals at the temporal sample *w*_0_ (the center of the time window of length *W* samples) for each dipole. By shifting the window *W* through the entire length of the MEG signal *T*, it is possible to retrieve the complete source signals.

It is worth noting that the choice of the value of *W* is crucial, as a larger window helps the algorithm, while a reduced length decreases both computational time and burden. In this paper, a good compromise has been found with 21 time samples; therefore, for all subsequent tests, *W* has been fixed at 21.

The training of a neural network involves the setting of parameters that affect the performance of the algorithm. More specifically, the training set, composed of 2 × 10^5^ input-output pairs, has been divided into mini-batches with 64 samples per batch (more details about the trainset composition are provided in the following sections). The adopted optimization strategy is the Adaptive Moment Estimation (Adam) with an initial learning rate of 10^−4^. Every iteration of the training process updates the weights of the network based on the values of the loss function, that in this case is the mean square error. Although the proposed architecture could be applied also to the EEG case, within this manuscript we focused only on MEG.

### 3.2 Dataset description

When adopting a learning-by-example approach, it is essential to consider an appropriate training dataset. Specifically, the dataset must strike a balance between generalization and accuracy.

In the context of training Deep-MEG, designing the dataset involves considering the forward problem described in Equation 10, which is closely related to the design of the matrices **Q**, **L**, and **M** from both geometric and electrical perspectives. For the MEG helmet, all simulations used to validate the proposed approach are conducted using an apparatus inspired by Rombetto et al. (2014), featuring *S* = 127 SQUID sensors. On the other hand, the number of dipoles *D* and their respective positions depend on the head model and the considered brain sources. To achieve this, the structural geometry of the head was derived from a real magnetic resonance image. MRI segmentation and brain extraction processes have been performed via the Matlab Toolbox Fieldtrip. By adopting a discretization step of 5 mm, the brain was parceled into 1,3467 sources. The Leadfield matrix **L** was computed assuming a single-shell head model (Cuffin and Cohen, 1977).

Regarding the brain signals, in all simulations we assumed that each active source produces a Gaussian-damped sine wave. This choice is common to other works in literature (Hossein et al., 2018; Liang et al., 2023). Specifically, each source signal *g*(*t*) is generated according to the following model:


(12) $$\begin{array}{l} g\left(t\right)=\sin\left(2\pi f_{0}t+\phi \right)\cdot \exp\left[-\frac{{\left(t-t_{0}\right)}^{2}}{\omega^{2}}\right]. \end{array}$$

where the parameters *t*_0_, ω, *f*_0_, and ϕ, which respectively denote the center and standard deviation of the Gaussian-damped wave, as well as the frequency and initial phase of the sine wave, vary across sources. We generated these parameters assuming a uniform random distribution, with ranges reported in Table 1. In particular, we choose to consider the so-called alpha band, nevertheless the extension of the approach to other frequency bands is trivial.

**Table 1:** Range of the values of the signal parameters exploited for the dataset generation (uniformly distributed).

	**Minimum value**	**Maximum value**
Gaussian center *t*_0_ [s]	0.05	0.3
Gaussian damping rate ω [s]	0.04	0.12
Sine wave frequency *f*_0_ [Hz]	8	14
Sine wave initial phase ϕ [rad]	0	2π

Each matrix **Q** (i.e. the dipole signals), and consequently each matrix **M** (i.e., the acquisition), spans a duration of 0.4 s, with a sampling frequency of 1 kHz, resulting in 400 samples for each example. Instead of the matrix **M**, the input of the network is a *W* samples window for each channel. We chose *W* = 21 as it corresponds to one period of a 50 Hz wave (20 ms at 1 kHz sampling frequency).

To enhance the generalization properties of Deep-MEG, the training set has been evenly split into examples of focal source activation and examples of extended source activation. Additionally, in order to increase the robustness of the approach, during the training phase additive white gaussian noise has added to MEG data in the sensor's space (SNR in the range [0–30]db). Further details about these scenarios are provided in the following section.

Throughout its entire training process, Deep-MEG required ~ 30h. The training environment utilized the Python library PyTorch (version 2.7.9 installed on Python 3.10.16) on a 24 GB NVIDIA Quadro RTX 6000 GPU. It is worth to note that a single training process has been carried out that includes all the considered simulated test cases. More precisely, an overall of 200,000 sensors/sources MEG data (inputs/outputs) pairs have been considered. The trainset has been divided into 5 sets, evenly split, including all the considered test cases (more details about the considered scenarios are provided in the following section). Regarding the test with real data, Deep-MEG has been re-trained considering the anatomy of the subjects while, the signals remain the same of the simulated case.

## 4 Results

This section presents the Deep-MEG performance evaluation. The proposed method has undergone testing in both simulated and real data, and the results have been compared to those obtained using conventional MEG source reconstruction approaches. More in detail, we analyzed the results in four different scenarios: single active dipole, multiple dipoles, extended sources, and real data.

### 4.1 Considered approaches

The current state-of-the-art in brain source reconstruction encompasses several algorithms. In this paper, we focused on four time-domain methods that, differently from the proposed algorithm, require the knowledge (or the estimation) of the covariance matrix.

- Linearly Constrained Minimum Variance (LCMV): a beamformer that scans predefined dipole locations, with a single dipole providing the spatial filter output for each location (Van Veen et al., 1997).
- Residual Variance (RV): this beamformer scans with a single dipole and computes the residual variance at each grid location (Scherg, 1990).
- Minimum Norm Estimation (MNE): it reconstructs all sources within the source space simultaneously by minimizing the difference between the real and predicted data subject to regularization (Fuchs et al., 1999).
- Exact Low-Resolution Electromagnetic Tomography (eLORETA): this method extends the assumption of MNE, using an iterative algorithm to account for depth bias in the sources. The solution achieves theoretically exact localization (Pascual-Marqui, 2007).

Within this manuscript, we exploited the Fieldtrip toolbox implementations of the cited approaches, working in Matlab environment (Oostenveld et al., 2010) in their standard configuration. It is worth to mention that the noise covariance matrix has been estimated via a Fieldtrip routine. For the reconstruction step, the Deep-MEG approaches assumed a 15 mm grid (which corresponds to *D* = 494 sources reconstructed), while all other approaches assumed a 5 mm discretization step. This choice was made to limit the computational burden of the DeepMEG algorithm.

The reconstruction grid of the state-of-the-art approach adopt a discretisation step of 5 mm.

### 4.2 Single focal source

To evaluate the performance of the proposed method, we initially assessed its capabilities in the scenario of a single dipole excitation. Here, the ground truth is represented by a single active dipole producing a signal *g*(*t*) (see Equation 12). Various metrics were employed to evaluate both localization and signal reconstruction performances:

- Distance of Localization Error (LDE) [mm]: this metric represents the average of the minumum euclidean distances between the true and the estimated sources.

Let ${\left\{Q_{k}\right\}}_{k=1}^{K}$ be the set of reference active sources and ${\left\{{\hat{Q}}_{h}\right\}}_{h=1}^{H}$ be the set of estimated active sources (where a source is considered “active” if its energy is higher than 50% of the maximum energy). The DLE is the mean value among *d*_1_, *d*_2_, ..., *d*_*K*_, where the generic *d*_*k*_ represents the minimum Euclidean distance between the position of reference active source *Q*_*k*_ and the set of position of the estimated active sources ${\hat{Q}}_{1},{\hat{Q}}_{2},...,{\hat{Q}}_{H}$, i.e.:


(13) $$\begin{array}{l} DLE=\frac{1}{K}\overset{K}{\underset{k=1}{\sum }}d_{k}=\frac{1}{K}\overset{K}{\underset{k=1}{\sum }}\underset{\begin{array}{c} h \end{array}}{\min}\left[||Q_{k}-{\hat{Q}}_{h}||\right] \end{array}$$

where *h* = 1, 2, ..., *H*.

- Active Volume (AV) [cm^3^]: AV measure the active volume of the brain. This value is a measure of localization spreading. For focal sources, the lower in the AV the more precise is the localization.
- Normalized Root Mean-Square Error (NRMSE): the NRMSE represents the average of the square-root mean-square error between the actual signal and the reconstructed one in the test set. Both signals have been normalized by the square root of their energy, and the quadratic difference has been computed between the envelopes of these normalized signals.

**Figure 3:**
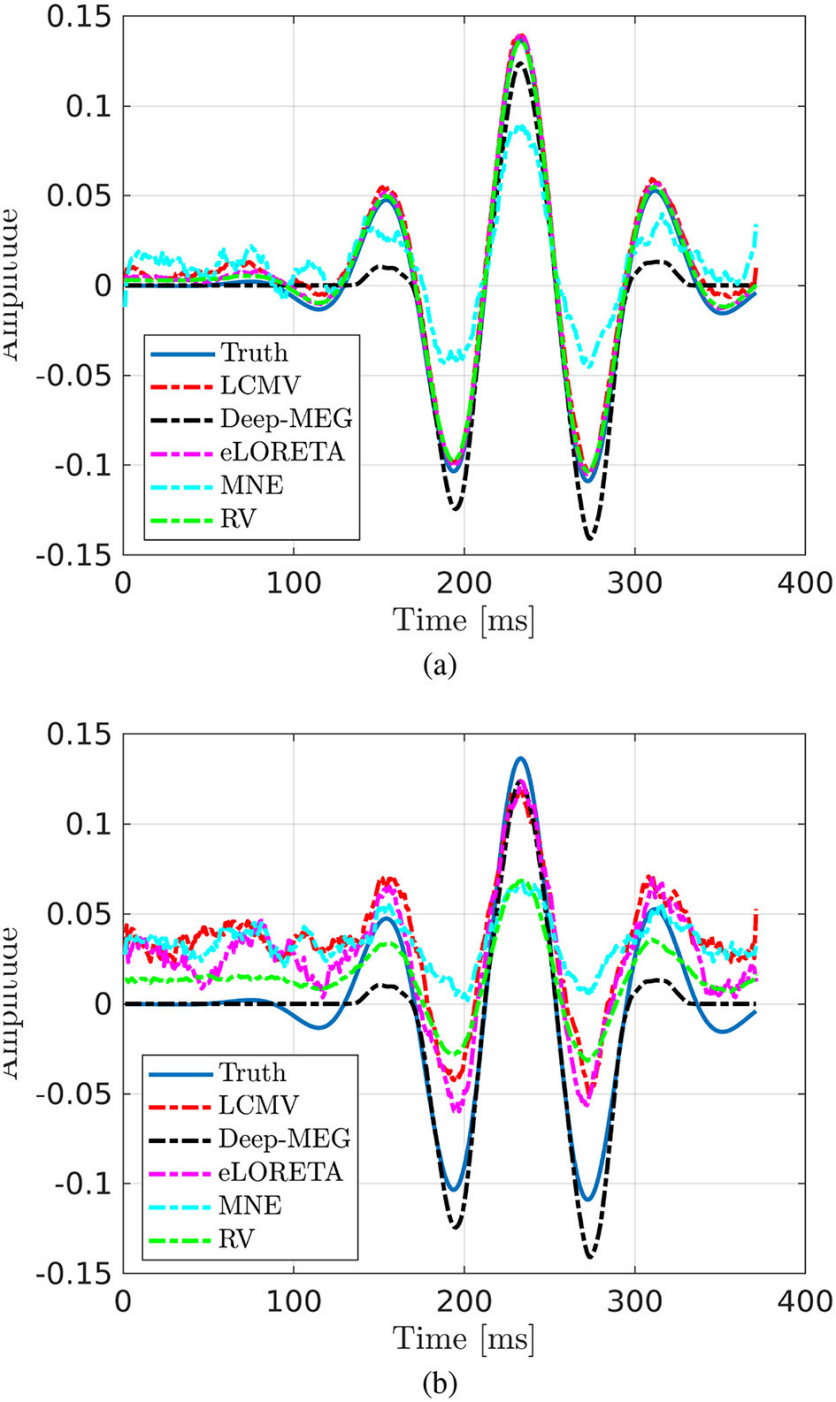
Signal reconstructions for a single focal case; SNR equal to **(a)** 30 dB and **(b)** 10 dB.

For both DLE and AV computation a dipole is considered active if its energy exceeds 50% of the maximum energy. The test set comprises 100 examples with random active dipole position and signal. To assess the algorithm's robustness, the data were corrupted by additive white gaussian noise (added to the sensor's space after the forward problem computation), reaching different SNR values (30, 20, 10 and 0 dB). In the following results for 30 and 10 dB cases are reported, for the complete analyses refer to the Appendix (Tables A1–A6). In Figure 3, an example of time reconstructions in case of high (Figure 3a) and low (Figure 3b) SNR in the single focal test is shown. In the high SNR case, all the approaches provides good estimation, with the MNE being the lower quality method. As expected, except for Deep-MEG, in the case of lower SNR, the performance of all the algorithms drop dramatically.

**Figure 4:**
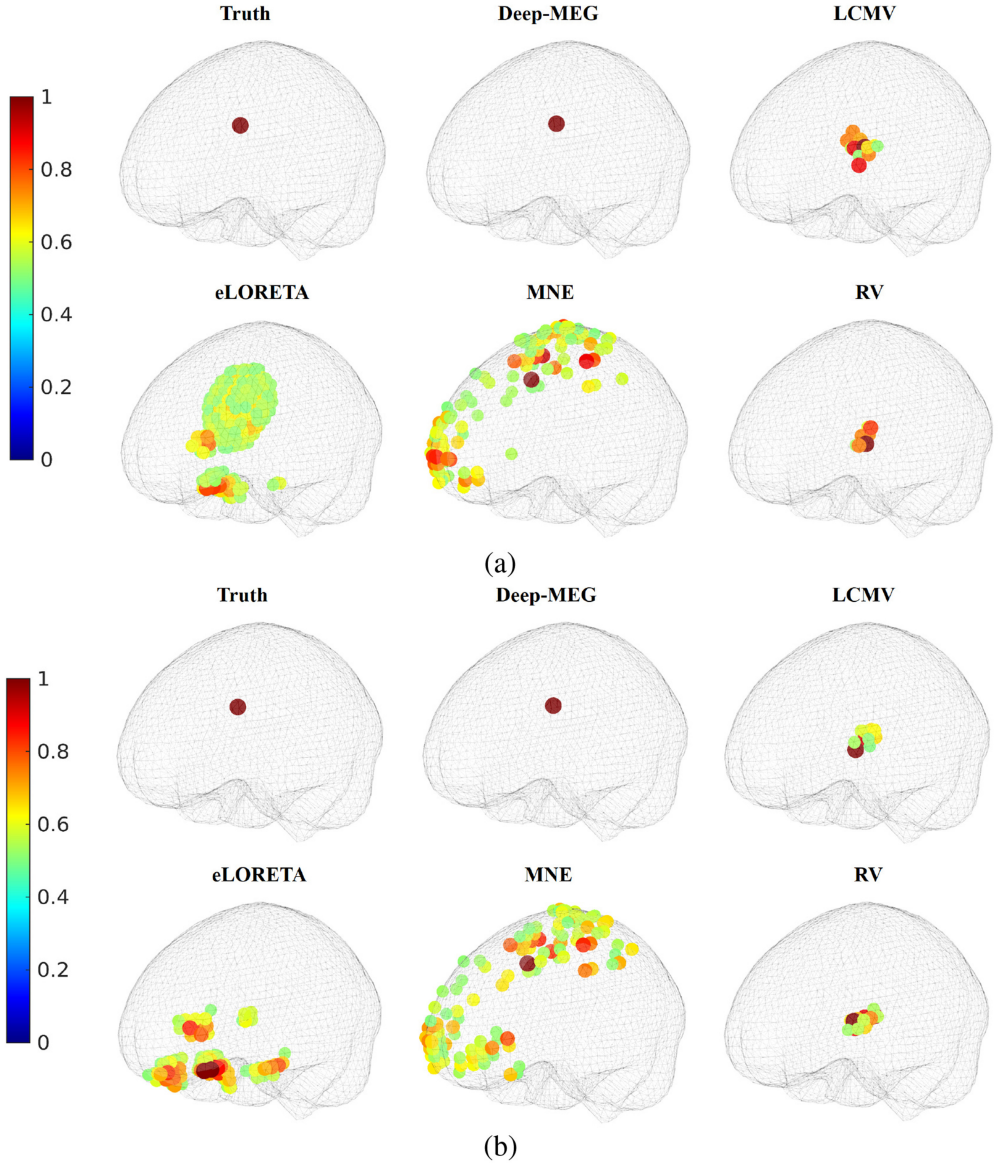
Source localization for the single focal case. Normalized power of the active regions for SNR equal to **(a)** 30 dB and **(b)** 10 dB are reported (only active regions are reported).

In Figure 4, the source localization results are reported. In the 30 dB case (Figure 4a), both Deep-MEG, LCMV and eLORETA correctly identify the active region, although eLORETA and LCMV reach a less sharp solution. As the SNR decreases (Figure 4b), the localization error of eLORETA and LCMV increases considerably, while the performance of the proposed method remains stable. Values reported in Table 2, that are related to the entire dataset, confirm such findings. These results demonstrate Deep-MEG robustness to noise in both localization and reconstruction tasks. Conversely, eLORETA exhibits good performance when the SNR is high, but its noise rejection appears to be ineffective. Furthermore, based on the AV values in the table, it is evident that the proposed method exhibits high accuracy.

**Table 2:** Single focal source case–Mean and standard deviation of Distance of Localization Error (DLE), Active Volume (AV) and Normalized Root Mean Square Error (NRMSE).

	**DLE [**mm**]**		**AV** [**cm**^**3**^**]**		**RMSE**	
	**30 dB**	**10 dB**	**30 dB**	**10 dB**	**30 dB**	**10 dB**
**Deep-MEG** (proposed)	8.96 ± 11.8	9.18 ± 11.7	7.48 ± 5.48	7.34 ± 5.14	0.45 ± 0.20	0.45 ± 0.21
**LCMV**	33.9 ± 24.4	46.2 ± 16.7	1.19 ± 0.67	1.12 ± 0.48	0.87 ± 0.09	0.87 ± 0.09
**eLORETA**	4.70 ± 14.7	54.2 ± 32.1	47.6 ± 27.5	15.6 ± 4.50	0.21 ± 0.25	0.85 ± 0.19
**MNE**	42.7 ± 21.2	48.4 ± 23.3	11.6 ± 2.50	5.96 ± 0.41	0.83 ± 0.10	0.85 ± 0.07
**RV**	46.9 ± 21.2	45.9 ± 16.1	1.03 ± 0.58	2.17 ± 0.19	0.26 ± 0.18	0.85 ± 0.10

Note that, for the single focal case, the real active area was about 0.13 cm^3^.

Regarding computational time, at execution time Deep-MEG processing typically takes a few hundred milliseconds for each test sample. In contrast, other approaches, which additionally involve covariance matrix estimations, require around 25 s for each test sample. All tests were conducted on a workstation equipped with an AMD Ryzen 9 3950X 16-Core CPU.

### 4.3 Multiple focal sources

**Figure 5:**
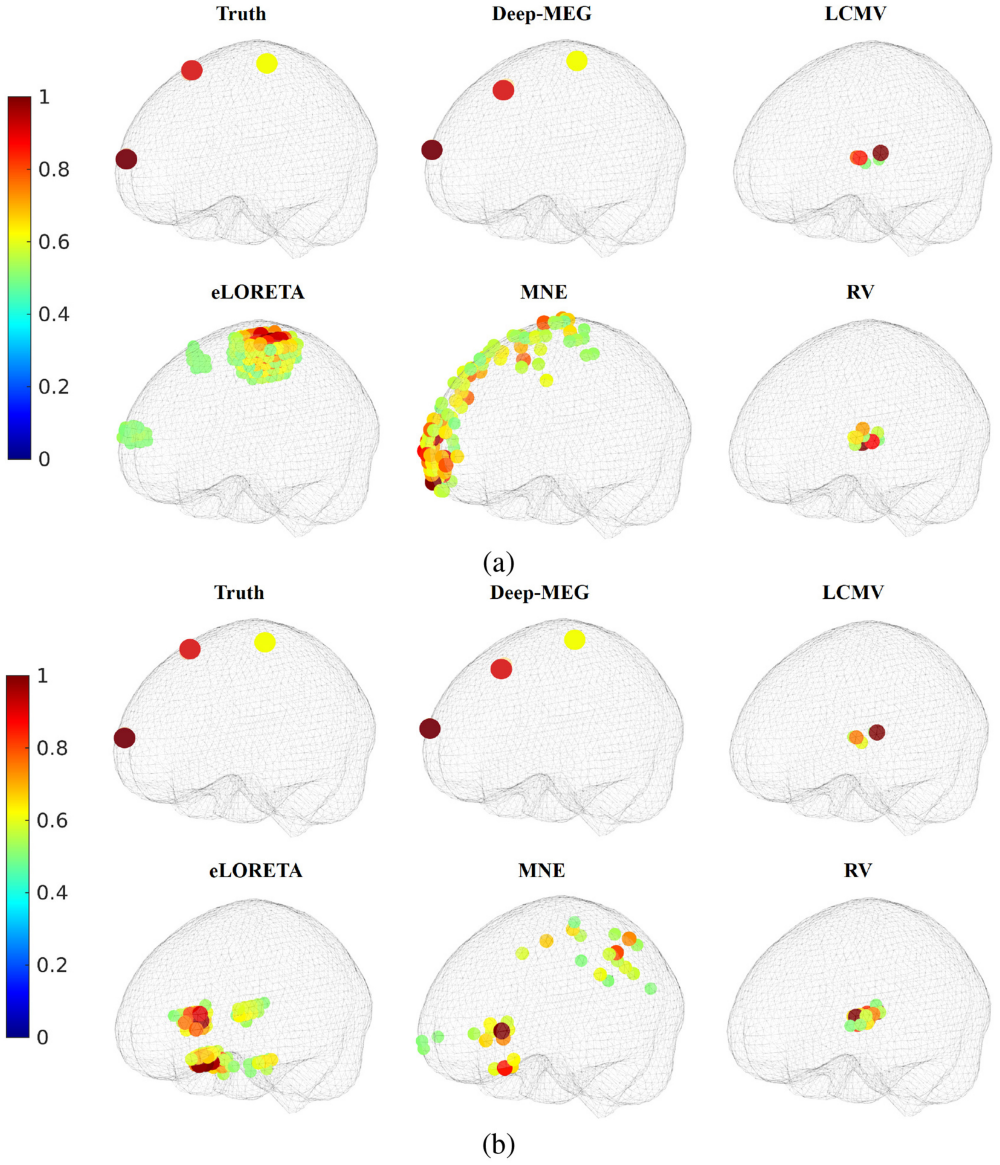
Source localization for the multiple focal case. Three simultaneous active sources have been considered. Normalized power of the active regions for SNR equal to **(a)** 30 dB and **(b)** 10 dB are reported.

To assess the spatial resolution of Deep-MEG, tests with different simultaneous active sources were conducted. We assumed two or three active focal sources, each producing a different signal *g*(*t*). For this analysis, we only focused on the source localization performances, as depicted in Figure 5. As in the previous case, Deep-MEG correctly identifies all active sources in both 30 dB and 10 dB cases. In the high SNR case, eLORETA exhibits a blurring effect, while in the 10 dB case, it fails to correctly locate the three active sources. MNE, in the 30 dB case presents a unique activation region, in the 30 dB case it fails to locate all the three regions. It can be noted that the other methods fail in localizing the active sources.

For a comprehensive analysis, all algorithms were tested using a test set of 100 cases per each of the two and three active source scenarios. Results of DLE and AV for the scenarios with multiple active sources are presented in Table 3. Deep-MEG demonstrates robustness to noise. Similarly to the single focal case, eLORETA performs well when the SNR is high, but its performance significantly deteriorates in the 10 dB SNR case. Although the DLE values for MNE are satisfactory, the AV values are substantially higher compared to Deep-MEG indicating a greater spreading of the active regions.

**Table 3:** Multiple focal sources case—Mean and standard deviation of the Distance of Localization Error (DLE), Active Volume (AV). Note that, for the double and triple focal case, the real active area was about, respectively, of 0.26 cm^3^ and 0.39 cm^3^.

	**2 active sources**				**3 active sources**			
	**DLE [mm]**		**AV [cm** ^3^ **]**		**DLE [mm]**		**AV [cm** ^3^ **]**	
	**30 dB**	**10 dB**	**30 dB**	**10 dB**	**30 dB**	**10 dB**	**30 dB**	**10 dB**
Deep-MEG	35.3 ± 18.1	35.2 ± 18.0	6.17 ± 3.91	6.33 ± 3.97	25.9 ± 14.1	26.0 ± 14.2	6.81 ± 3.55	7.03 ± 3.85
LCMV	57.9 ± 10.6	58.7 ± 8.66	1.05 ± 0.98	1.03 ± 0.38	58.3 ± 7.63	58.7 ± 6.83	0.94 ± 0.50	0.98 ± 0.37
eLORETA	25.2 ± 17.8	70.8 ± 19.6	24.2 ± 16.5	14.7 ± 2.32	21.0 ± 14.4	67.4 ± 13.1	34.1 ± 18.0	14.3 ± 0.71
MNE	32.5 ± 16.8	35.6 ± 12.1	12.8 ± 0.95	6.22 ± 0.31	22.0 ± 10.1	42.0 ± 9.34	12.8 ± 0.85	6.03 ± 0.34
RV	59.3 ± 8.00	57.3 ± 6.83	1.19 ± 0.50	2.22 ± 0.07	58.1 ± 6.66	58.7 ± 6.82	1.36 ± 0.46	2.15 ± 0.15

### 4.4 Extended sources

The final simulated scenario involves extended active sources, where an entire sub-volume of the brain generates a unique realization of *g*(*t*). Specifically, the cases of one and of two extended active sources were considered.

**Figure 6:**
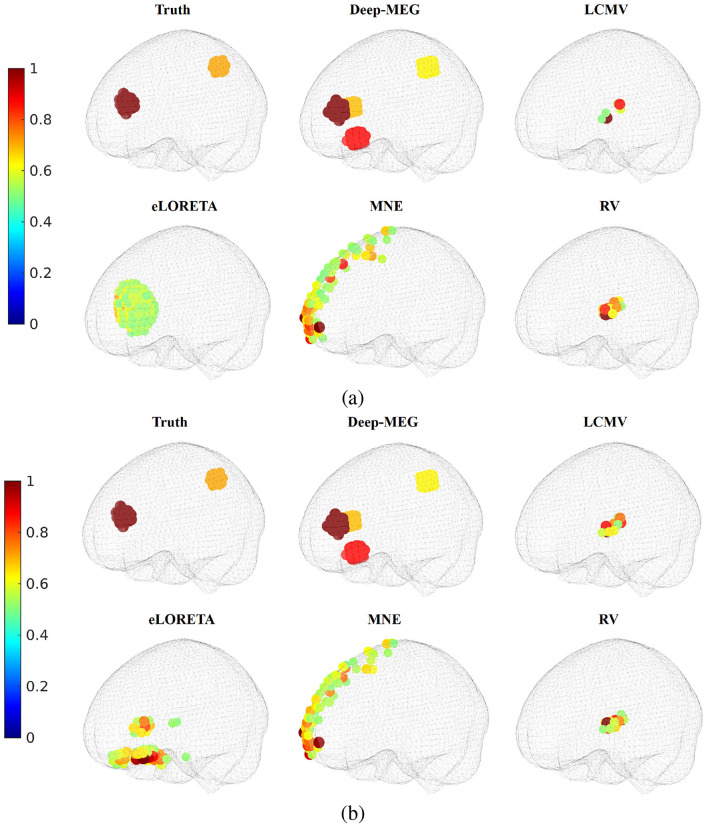
Source localization for the extended source case. Normalized power of the active regions for SNR equal to **(a)** 30 dB and **(b)** 10 dB.

In Figure 6 an example of source localization in the scenario with two extended sources is reported. In the high SNR case, eLoreta correctly identify one of the two active regions with a slightly spreading. The proposed Deep-MEG correctly identifies both regions as distinct zones presenting two additional active regions, the other approches fail to locate both regions. In the 10 dB SNR case, Deep-MEG maintain its performance, whereas eLoreta exhibits a deterioration.

The localization performances were measured in terms of Intersection over Union (IoU) and DLE. The IoU indicates the ratio between the intersection of active volumes of the ground truth and reconstructions, and the union of such volumes.

Table 4 reports the DLE and the IoU metrics for a test set of 100 cases with single active extended region and 100 with two active extended areas. The volume of an active region has been set between of 14 and 44 cm^3^.

**Table 4:** Extended sources case—Mean and standard deviation of Distance of Localization Error (DLE), Intersection over Union (IoU).

	**Single extended source**				**Double extended source**			
	**DLE [mm]**		**IoU [%]**		**DLE [mm]**		**IoU [%]**	
	**30 dB**	**10 dB**	**30 dB**	**10 dB**	**30 dB**	**10 dB**	**30 dB**	**10 dB**
Deep-MEG	10.4 ± 12.5	10.8 ± 11.9	12.0 ± 13.3	10.8 ± 12.3	30.3 ± 16.3	30.9 ± 15.3	9.98 ± 10.1	9.82 ± 9.71
LCMV	40.8 ± 24.7	50.8 ± 16.7	1.99 ± 4.36	0.50 ± 2.75	44.1 ± 15.2	48.6 ± 10.5	1.13 ± 2.93	0.05 ± 0.54
eLORETA	5.49 ± 18.6	55.6 ± 34.9	11.8 ± 8.28	1.94 ± 5.13	20.8 ± 15.1	58.5 ± 20.7	14.5 ± 7.96	0.62 ± 2.76
MNE	57.6 ± 30.9	47.7 ± 28.8	0.06 ± 0.32	0.12 ± 0.47	61.3 ± 23.6	48.2 ± 10.1	0.12 ± 0.60	0.16 ± 0.59
RV	52.6 ± 14.9	51.5 ± 14.2	0.11 ± 0.70	0.13 ± 0.87	49.4 ± 10.6	48.2 ± 10.1	0.02 ± 0.23	0.07 ± 0.74

In these scenarios, eLORETA performs better than the other methods for an SNR equal to 30 dB. However, when the SNR decreases, while Deep-MEG maintains its performance, eLORETA experiences a significant decline in both DLE and IoU.

### 4.5 Depth analysis

One of the novel aspects of the proposed approach is its ability to locate and reconstruct deep sources. In this subsection, the performance of Deep-MEG in the case of deep sources is evaluated. Specifically, for the single focal case, the test set was subdivided into two sets based on the distance between the active source of each example and a reference point called the 'deep point.' The deep point is defined as the brain dipole with the greatest mean distance from the MEG sensors. The 50 test examples presenting an active source with the shortest distance from the deep point constitute the deep sources subset, while the remaining 50 form the cortical sources subset. Table 5 shows the performance of the considered solutions in terms of DLE, AV, and NRMSE for both deep and cortical sources in the single focal source scenario (note that the SNR was fixed at 30dB for these analyses). From the table, it is possible to note that the localization performance depends on the depth of the source. In particular, Deep-MEG, eLoreta and MNE exhibit a reduction of the precision (the decrease of Deep-MEG is lower compared to eLoreta and MNE), while LCMV and RV increase their precision in deep source cases. Regarding the AV parameter, LCMV, MNE and RV remain quite stable while Deep-MEG and eLoreta present a greater blurring effect in deep source cases (also in this case Deep-MEG is more stable than eLoreta). Finally, regarding RMSE, LCMV and MNE remains stable, Deep-MEG and RV present a slightly decrease of performance while eLoreta dramatically drop its reconstruction properties.

**Table 5:** Mean values and standard deviation of Distance of Localization Error (DLE), Active Volume (AV) and Normalized Root Mean Square Error (NRMSE) for Deep-MEG reconstructions.

	**DLE [mm]**		**AV** [**cm**^**3**^]		**NRMSE**	
	**Cortical**	**Deep**	**Cortical**	**Deep**	**Cortical**	**Deep**
Deep-MEG	5.59 ± 6.10	12.3 ± 14.9	5.71 ± 3.70	9.24 ± 6.50	0.36 ± 0.18	0.53 ± 0.19
LCMV	48.8 ± 18.5	18.9 ± 20.2	1.18 ± 1.38	1.19 ± 1.38	0.87 ± 0.10	0.87 ± 0.09
eLORETA	0 ± 0	9.40 ± 19.8	33.4 ± 13.7	62.0 ± 30.5	0.07 ± 0.04	0.35 ± 0.29
MNE	35.5 ± 22.8	52.6 ± 20.4	12.7 ± 11.2	10.6 ± 12.6	0.83 ± 0.11	0.86 ± 0.06
RV	54.1 ± 11.1	39.7 ± 18.2	1.17 ± 1.25	0.90 ± 1.25	0.19 ± 0.12	0.33 ± 0.20

Single focal source case with examples divided in deep and cortical sources [SNR = 30 dB].

### 4.6 Real MEG data

In this final analysis, the algorithm was tested using real data from the “OpenNEURO” database, under accession number ds000117 (Henson et al., 2011; Wakeman and Henson, 2015). The acquisition involved the simultaneous acquisition of MEG and EEG signals from subjects performing a visual recognition task involving famous, unfamiliar, and scrambled faces. For our testing purposes, the MEG data of a single subject were considered. The acquisition system was an Elekta-Neuromag VectorView with 306 sensors, and the sampling frequency was 1,100 Hz.

The OpenNEURO database also provided the magnetic resonance images of the subjects, and we exploited them to produce the digital brain volume. Regarding the forward problem, the brain was discretized with a 5 mm step, resulting in 12,211 dipoles and the Leadfield matrix was estimated. Deep-MEG was trained with the same simulated signals described in 3. As in the previous cases, for the inverse problem, the brain was discretized with a 15 mm step, resulting in 453 dipoles for Deep-MEG while the other approaches adopted the discretisation step of 5 mm.

The raw MEG signals underwent preprocessing using the Maxfilter Signal-Source Separation algorithm (SSS) (Taulu and Kajola, 2005; Taulu and Simola, 2006). The SSS algorithm is responsible for separating the magnetic signals originating from the brain from those originating outside. It removes noise, detects bad channels, and realigns data after movements.

**Figure 7:**
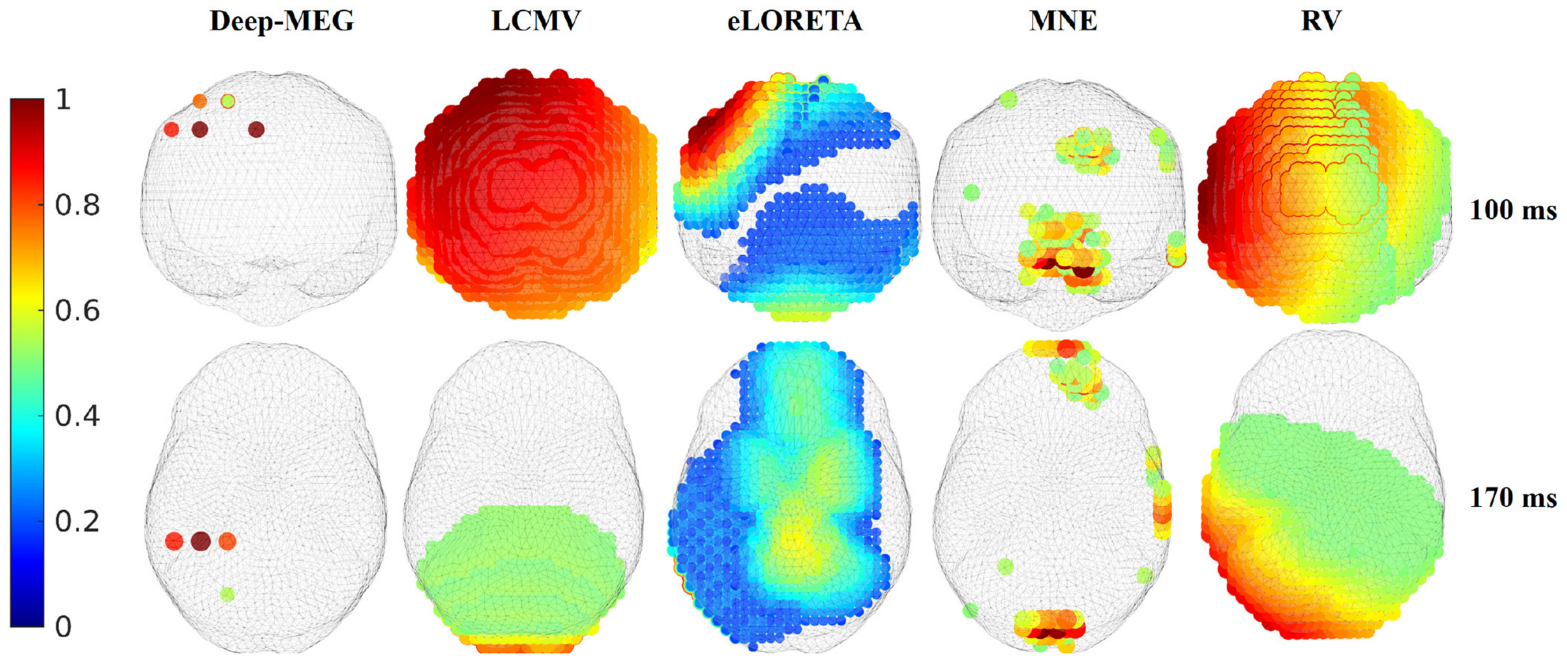
Results of the MEG source imaging for the real data related to the face recognition task. The instantaneous powers at 100 and 170 ms after the visual stimulus are reported.

Figure 7 illustrates source imaging of the MEG signals for the face recognition task. The images depict the instantaneous power of each dipole 100 ms and 170 ms after the presentation of a famous face stimulus. In the 100 ms post stimulus case (first line of Figure 7), activations in the right occipital area have been found by Liang et al. (2023); Xu (2005). The same active area has been reconstructed by Deep-MEG and all the considered approaches but MNE, although each method with a different spread. Moving to the 170 ms post stimulus case (second line of Figure 7), an activation of the right fusiform area is expected to emerge, according to literature. Looking at the results, Deep-MEG clearly shows this activation, as does eLORETA although much more widespread, while the other methods show more occipital activations (LCMV and MNE) or which insist on almost all the parenchyma (RV).

## 5 Discussion

In this paper, we address the problem of brain source localization and reconstruction from MEG data using a novel deep learning approach: Deep-MEG. This method introduces a hybrid artificial neural network composed of two main components. The first component is a cascade of convolutional layers that extracts temporal information, while the second consists of fully connected layers responsible for spatial information extraction. Deep-MEG has been evaluated across various scenarios involving both single and multiple focal and extended sources. Its performance has been compared with existing methods from the literature, demonstrating its effectiveness—particularly under low signal-to-noise ratio (SNR) conditions. Deep-MEG has been also tested exploiting real MEG data obtained by a public dataset. The proposed solution shows results comparable to literature. Although this study focuses on MEG data, Deep-MEG essentially learns the mapping between measurements on the scalp and neuronal activity in the brain. Therefore, it can also be applied to EEG data without requiring structural changes. However, there are some limitations worth noting. The training phase of Deep-MEG takes approximately 30 h, and the computational demands are significant, especially with higher resolution grids. Furthermore, the model is subject-dependent; any change in the MEG device or the subject requires retraining the network. In such cases, fine-tuning strategies can help reduce retraining time. Given these considerations, Deep-MEG is recommended for applications where accurate source localization is critical. In situations where this level of precision is not necessary, traditional methods such as eLoreta may suffice, offering reduced computational time.

## 6 Conclusion

Within this manuscript the Deep-MEG methodology is presented and tested. The algorithm is able to perform MEG source localization and reconstruction by means of neural networks and deep learning. More in detail, the proposed method consists of an hybrid neural network that jointly exploits convolutional layers for the extraction of the temporal features and fully connected layers for the spatial identification of the brain activations.

The algorithm has been tested in both simulated and real scenarios, showing interesting performances both in localization and signal estimation tasks, even with low values of SNR, demonstrating good noise robustness and spatial discrimination of the proposed approach. More in detail, the good performances characterized all the considered test cases, with the proposed methodology being able to correctly handle both the multiple focal and the extended sources. In addition, the analysis on real data yielded results consistent with the existing literature.

In the future, we will focus on generalizing the method across different head models and evaluate the impact of Deep-MEG reconstructions on brain functional network analyses.

## Data Availability

The raw data supporting the conclusions of this article will be made available by the authors, without undue reservation.

## References

[B1] Ambrosanio M. Franceschini S. Baselice F. Pascazio V. (2020). “Machine learning for microwave imaging,” in 14th European Conference on Antennas and Propagation, EuCAP 2020 (Copenhagen). 10.23919/EuCAP48036.2020.9136081

[B2] Ambrosanio M. Franceschini S. Pascazio V. Baselice F. (2022). An end-to-end deep learning approach for quantitative microwave breast imaging in real-time applications. Bioengineering 9:651. 10.3390/bioengineering9110651 36354562 PMC9687617

[B3] Autorino M. M. Franceschini S. Ambrosanio M. Pascazio V. Baselice F. (2024). Intra voxel analysis in magnetic resonance imaging via deep learning. Int. J. Imaging Syst. Technol. 34:e22977. 10.1002/ima.2297740112509

[B4] Baillet S. Mosher J. Leahy R. (2001). Electromagnetic brain mapping. IEEE Signal Process. Mag. 18:14–30. 10.1109/79.962275

[B5] Cai C. Long Y. Ghosh S. Hashemi A. Gao Y. Diwakar M.. (2023). Bayesian adaptive beamformer for robust electromagnetic brain imaging of correlated sources in high spatial resolution. IEEE Trans. Med. Imaging. 42. 10.1109/TMI.2023.3256963 37028341

[B6] Cohen D. (1972). Magnetoencephalography: detection of the brain's electrical activity with a superconducting magnetometer. Science 175, 664–666. 10.1126/science.175.4022.664 5009769

[B7] Cuffin B. Cohen D. (1977). Magnetic fields of a dipole in special volume conductor shapes. IEEE Trans. Biomed. Eng. 24, 372–381. 10.1109/TBME.1977.326145 881208

[B8] Ermer J. Mosher J. Huang M. Leahy R. (2000). Paired MEG data set source localization using recursively applied and projected (RAP) music. IEEE Trans. Biomed. Eng. 47, 1248–1260. 10.1109/10.867959 11008426

[B9] Ferraioli G. Pascazio V. Vitale S. (2019). “A novel cost function for despeckling using convolutional neural networks,” in 2019 Joint Urban Remote Sensing Event (JURSE) (Vannes), 1–4. 10.1109/JURSE.2019.8809042

[B10] Franceschini S. Ambrosanio M. Baselice F. Pascazio V. (2021). “Neural networks for inverse problems: the microwave imaging case,” in 2021 15th European Conference on Antennas and Propagation (EuCAP), 1–5. 10.23919/EuCAP51087.2021.941131736428846

[B11] Fuchs M. Wagner M. Köhler Wischmann, H. (1999). Linear and nonlinear current density reconstructions. J. Clin. Neurophysiol. 16, 267–295. 10.1097/00004691-199905000-00006 10426408

[B12] Goodfellow I. Bengio Y. Courville A. (2016). Deep Learning. MIT press.

[B13] Hecker L. Rupprecht R. Tebartz Van Elst L. Kornmeier J. (2021). ConvDip: A convolutional neural network for better EEG source imaging. Front. Neurosci. 15:569918. 10.3389/fnins.2021.569918 34177438 PMC8219905

[B14] Henson R. N. Wakeman D. G. Litvak V. Friston K. J. (2011). A parametric empirical Bayesian framework for the EEG/MEG inverse problem: generative models for multi-subject and multi-modal integration. Front. Hum. Neurosci. 5:76. 10.3389/fnhum.2011.00076 21904527 PMC3160752

[B15] Hincapié A. Kujala J. Mattout J. Daligault S. Delpuech C. Mery D.. (2016). MEG connectivity and power detections with minimum norm estimates require different regularization parameters. Comput. Intell. Neurosci. 2016:3979547. 10.1155/2016/3979547 27092179 PMC4820599

[B16] Hossein H. Seyed A. Sohrabpour A. Akçakaya M. He B. (2018). Electromagnetic brain source imaging by means of a robust minimum variance beamformer. IEEE Trans. Biomed. Eng. 65, 2365–2374. 10.1109/TBME.2018.2859204 30047869 PMC7934089

[B17] Jonmohamadi Y. Poudel G. Innes C. Weiss D. Krueger R. Jones R. (2014). Comparison of beamformers for EEG source signal reconstruction. Biomed. Signal Process. Control 14, 175–188. 10.1016/j.bspc.2014.07.014

[B18] Jun Y. Eom T. Kim Y. Chung S. Lee I. Kim J. (2019). Changes in background electroencephalographic activity in benign childhood epilepsy with centrotemporal spikes after oxcarbazepine treatment: a standardized low-resolution brain electromagnetic tomography (sLORETA) study. BMC Neurol. 19:3. 10.1186/s12883-018-1228-8 30606133 PMC6317234

[B19] Kleiner R. Koelle D. Ludwig F. Clarke J. (2004). Superconducting quantum interference devices: state of the art and applications. Proc. IEEE 92, 1534–1548. 10.1109/JPROC.2004.833655

[B20] LeCun Y. Bengio Y. Hinton G. (2015). Deep learning. Nature 521, 436–444. 10.1038/nature14539 26017442

[B21] Liang J. Yu Z. L. Gu Z. Li Y. (2023). Electromagnetic source imaging with a combination of sparse bayesian learning and deep neural network. IEEE Trans. Neural Syst. Rehabil. Eng. 31, 2338–2348. 10.1109/TNSRE.2023.3251420 37028383

[B22] Litjens G. Kooi T. Bejnordi B. E. Setio A. A. A. Ciompi F. Ghafoorian M.. (2017). A survey on deep learning in medical image analysis. Med. Image Anal. 42, 60–88. 10.1016/j.media.2017.07.005 28778026

[B23] Malmivuo J. Plonsey R. (1995). Bioelectromagnetism: Principles and Applications of Bioelectric and Biomagnetic Fields. Oxford University Press. 10.1093/acprof:oso/9780195058239.001.0001

[B24] Moiseev A. Herdman A. Ribary U. (2022). Subspace based multiple constrained minimum variance (SMCMV) beamformers. Biomed. Signal Process. Control 71:103124. 10.1016/j.bspc.2021.103124

[B25] Mosher J. Leahy R. (1998). Recursive MUSIC: a framework for EEG and MEG source localization. IEEE Trans. Biomed. Eng. 45, 1342–1354. 10.1109/10.725331 9805833

[B26] Mosher J. Lewis P. Leahy R. (1992). Multiple dipole modeling and localization from spatio-temporal MEG data. IEEE Trans. Biomed. Eng. 39, 541–557. 10.1109/10.141192 1601435

[B27] Nunes A. S. Moiseev A. Kozhemiako N. Cheung T. Ribary U. Doesburg S. (2020). Multiple constrained minimum variance beamformer (MCMV) performance in connectivity analyses. Neuroimage 208:116386. 10.1016/j.neuroimage.2019.116386 31786165

[B28] Oostenveld R. Fries P. Maris E. Schoffelen J. (2010). Fieldtrip: open source software for advanced analysis of MEG, EEG, and invasive electrophysiological data. Comput. Intell. Neurosci. 2011:156869. 10.1155/2011/156869 21253357 PMC3021840

[B29] Pantazis D. Adler A. (2021). MEG source localization via deep learning. Sensors 21:4278. 10.3390/s21134278 34206620 PMC8271934

[B30] Pascual-Marqui R. D. (2007). Discrete, 3D distributed, linear imaging methods of electric neuronal activity. Part 1: exact, zero error localization. arXiv. 10.48550/arXiv.0710.3341

[B31] Pereira S. Pinto A. Alves V. Silva C. A. (2016). Brain tumor segmentation using convolutional neural networks in MRI images. IEEE Trans. Med. Imaging 35, 1240–1251. 10.1109/TMI.2016.2538465 26960222

[B32] Rombetto S. Granata C. Vettoliere A. Russo M. (2014). Multichannel system based on a high sensitivity superconductive sensor for magnetoencephalography. Sensors 14, 12114–12126. 10.3390/s140712114 25006995 PMC4168467

[B33] Rucco R. Baselice F. Ambrosanio M. Vettoliere A. Sorrentino P. Riccio M. P.. (2020). Brain connectivity study by multichannel system based on superconducting quantum magnetic sensors. Eng. Res. Express 2:15038. 10.1088/2631-8695/ab7869

[B34] Scherg M. (1990). Fundamentals of dipole source potential analysis. Adv. Audiol. 6, 40–69.

[B35] S. E. Robinson J. V. (1999). “Functional neuroimaging by synthetic aperture magnetometry (SAM),” in Recent Advances in Biomagnetism (Sendai), 302–305.

[B36] Senaratne D. Tellambura C. (2010). “Generalized singular value decomposition for coordinated beamforming in MIMO systems,” in 2010 IEEE Global Telecommunications Conference GLOBECOM 2010 (Miami, FL: IEEE), 1–6. 10.1109/GLOCOM.2010.5684109

[B37] Sun R. Zhang W. Bagić A. He B. (2023). Deep learning based source imaging provides strong sublobar localization of epileptogenic zone from meg interictal spikes. Neuroimage 281:120366. 10.1016/j.neuroimage.2023.120366 37716593 PMC10771628

[B38] Taulu S. Kajola M. (2005). Presentation of electromagnetic multichannel data: the signal space separation method. J. Appl. Phys. 97:124905. 10.1063/1.1935742

[B39] Taulu S. Simola J. (2006). Spatiotemporal signal space separation method for rejecting nearby interference in meg measurements. Phys. Med. Biol. 51:1759. 10.1088/0031-9155/51/7/008 16552102

[B40] Teramoto A. Fujita H. Yamamuro O. Tamaki T. (2016). Automated detection of pulmonary nodules in PET/CT images: Ensemble false-positive reduction using a convolutional neural network technique. Med. Phys. 43, 2821–2827. 10.1118/1.4948498 27277030

[B41] Van Veen B. Van Drongelen W. Yuchtman M. Suzuki A. (1997). Localization of brain electrical activity via linearly constrained minimum variance spatial filtering. IEEE Trans. Biomed. Eng. 44, 867–880. 10.1109/10.623056 9282479

[B42] Wakeman D. G. Henson R. N. (2015). A multi-subject, multi-modal human neuroimaging dataset. Sci. Data 2:150001. 10.1038/sdata.2015.1 25977808 PMC4412149

[B43] Wei C. Lou K. Wang Z. Zhao M. Mantini D. Liu Q. (2021). Edge sparse basis network: a deep learning framework for EEG source localization. arXiv. 10.1109/IJCNN52387.2021.9533968

[B44] Westner B. Dalal S. Gramfort A. Litvak V. Mosher J. Oostenveld R.. (2022). A unified view on beamformers for M/EEG source reconstruction. Neuroimage 246:118789. 10.1016/j.neuroimage.2021.118789 34890794

[B45] Woolrich M. Hunt L. Groves A. Barnes G. (2011). MEG beamforming using Bayesian PCA for adaptive data covariance matrix regularization. Neuroimage 57, 1466–1479. 10.1016/j.neuroimage.2011.04.041 21620977 PMC4894461

[B46] Xu Y. (2005). Revisiting the role of the fusiform face area in visual expertise. Cereb. Cortex 15, 1234–1242. 10.1093/cercor/bhi006 15677350

[B47] Yu Z. Kachenoura A. Jeannès R. L. B. Shu H. Berraute P. Nica A.. (2024). Electrophysiological brain imaging based on simulation-driven deep learning in the context of epilepsy. Neuroimage 285:120490. 10.1016/j.neuroimage.2023.120490 38103624

[B48] Zemouri R. Zerhouni N. Racoceanu D. (2019). Deep learning in the biomedical applications: recent and future status. Appl. Sci. 9:1526. 10.3390/app9081526

