# Supplementary material for: Magnetoencephalographic source localization and reconstruction via deep learning
Source: Front Neurosci. 2025 Jul 21;19:1578473. doi: 10.3389/fnins.2025.1578473 (PMC12391924; doi:10.3389/fnins.2025.1578473)
Supplement: Supplementary file 1 [file Table_1.pdf]

Magnetoencephalographic Source Localization and Reconstruction via Deep Learning – Supplementary material

APPENDIX - COMPLETE RESULTS

In this appendix numerical results for all the tested cases and SNRs are reported (from Table A.1 to Table A.6).

Table A.1. Single focal source case - Mean and standard deviation of Distance of Localization Error (DLE) and Active Volume (AV) for different SNR values.

	DLE [mm]				AV [cm³]			
	30 dB	20 dB	10 dB	0 dB	30 dB	20 dB	10 dB	0 dB
**Deep-MEG** (proposed)	8.96 ± 11.8	8.91 ± 11.8	9.18 ± 11.7	11.8 ± 17.9	7.47 ± 5.47	7.41 ± 5.31	7.33 ± 5.13	7.67 ± 5.80
**LCMV**	33.9 ± 24.4	44.2 ± 18.8	46.2 ± 16.7	46.4 ± 16.3	1.18 ± 0.67	1.03 ± 0.58	1.03 ± 0.38	1.42 ± 0.39
**eLORETA**	4.70 ± 14.7	26.7 ± 31.7	54.2 ± 32.1	61.9± 29.1	47.6 ± 27.5	28.3 ± 18.5	15.6 ± 4.50	14.2 ± 0.84
**MNE**	42.7 ± 21.2	44.0 ± 18.9	48.4 ± 23.3	41.7 ± 21.2	11.6 ± 2.50	8.05 ± 2.63	5.96 ± 0.41	16.0 ± 0.88
**RV**	46.9 ± 16.7	46.0 ± 16.4	45.9 ± 16.1	45.9 ± 16.1	1.03 ± 0.58	1.81 ± 0.57	2.17 ± 0.19	2.25 ± 0.01

Table A.2. Single focal source case - Mean and standard deviation of Normalised Root Mean Square Error (NRMSE) for different SNR values.

	Normalised Root Mean Square Error			
	30 dB	20 dB	10 dB	0 dB
**Deep-MEG** (proposed)	0.45 ± 0.20	0.44 ± 0.20	0.46 ± 0.21	0.51 ± 0.25
**LCMV**	0.87 ± 0.09	0.87 ± 0.09	0.87 ± 0.09	0.87 ± 0.09
**eLORETA**	0.21 ± 0.25	0.57 ± 0.35	0.85 ± 0.19	0.90 ± 0.10
**MNE**	0.83 ± 0.10	0.85 ± 0.08	0.85 ± 0.07	0.89 ± 0.06
**RV**	0.26 ± 0.18	0.62 ± 0.22	0.85 ± 0.10	0.85 ± 0.09

Table A.3. Double focal sources case - Mean and standard deviation of Distance of Localisation Error (DLE) and Active Volume (AV) for different SNR values.

	DLE [mm]				AV [cm³]			
	30 dB	20 dB	10 dB	0 dB	30 dB	20 dB	10 dB	0 dB
**Deep-MEG**	35.3 ± 18.1	35.3 ± 18.1	35.2 ± 18.0	35.7 ± 18.3	6.17 ± 3.91	6.23 ± 3.88	6.33 ± 3.97	6.22 ± 3.88
**LCMV**	57.9 ± 10.6	58.7 ± 9.20	58.7 ± 8.66	58.4 ± 8.47	9.48 ± 5.37	0.94 ± 0.45	1.03 ± 0.38	1.28 ± 0.28
**eLORETA**	25.2 ± 17.8	24.9 ± 18.6	70.8 ± 19.6	72.6 ± 17.5	24.2 ± 16.5	25.7 ± 15.8	14.7 ± 2.32	14.2 ± 0.81
**MNE**	32.5 ± 16.8	26.6 ± 14.2	35.6 ± 12.1	29.6 ± 15.9	12.8 ± 0.95	13.2 ± 2.83	6.22 ± 0.31	12.4 ± 0.17
**RV**	59.3 ± 8.00	58.4 ± 8.26	57.3 ± 8.23	57.0 ± 9.18	1.19 ± 0.50	1.80 ± 0.39	2.22 ± 0.07	2.25 ± 0.00

Table A.4. Triple focal sources case - Mean and standard deviation of Distance of Localisation Error (DLE) and Active Volume (AV) for different SNR values.

	DLE [mm]				AV [cm^3]			
	30 dB	20 dB	10 dB	0 dB	30 dB	20 dB	10 dB	0 dB
**Deep-MEG**	25.9 ± 14.1	26.2 ± 14.0	26.0 ± 14.2	28.0 ± 14.6	6.81 ± 3.55	6.81 ± 3.57	7.03 ± 3.85	7.10 ± 3.82
**LCMV**	58.3 ± 7.63	58.4 ± 7.01	58.7 ± 6.83	59.7 ± 6.88	0.94 ± 0.50	0.95 ± 0.43	0.98 ± 0.37	1.04 ± 0.28
**eLORETA**	21.0 ± 14.4	24.2 ± 17.1	67.4 ± 13.1	68.6 ± 11.9	34.1 ± 18.0	30.1 ± 13.6	14.3 ± 1.96	14.1 ± 0.36
**MNE**	22.0 ± 10.1	22.1 ± 9.44	42.0 ± 9.34	21.1 ± 9.59	12.8 ± 0.95	13.6 ± 2.03	6.03 ± 0.34	13.5 ± 0.09
**RV**	58.1 ± 6.66	58.7 ± 6.89	58.7 ± 6.82	58.4 ± 6.66	1.36 ± 0.46	1.96 ± 0.43	2.15 ± 0.15	2.25 ± 0.00

Table A.5. Single extended sources case - Mean and standard deviation of Distance of Localisation Error (DLE) and Intersection over Union (IoU) for different SNR values.

	DLE [mm]				IoU [%]			
	30 dB	20 dB	10 dB	0 dB	30 dB	20 dB	10 dB	0 dB
**Deep-MEG**	10.4 ± 12.5	10.2 ± 11.9	10.8 ± 11.9	17.8 ± 24.3	9.98 ± 10.1	11.8 ± 12.8	10.8 ± 12.3	9.69 ± 12.0
**LCMV**	40.8 ± 24.7	48.1 ± 20.3	50.8 ± 16.7	52.4 ± 14.4	1.99 ± 4.36	0.92 ± 3.10	0.50 ± 2.75	0.10 ± 0.64
**eLORETA**	5.49 ± 18.6	22.9 ± 33.3	55.6 ± 34.9	62.5 ± 31.6	11.8 ± 8.28	8.93 ± 9.64	1.94 ± 5.13	0.51 ± 2.25
**MNE**	57.6 ± 30.9	50.8 ± 29.9	47.7 ± 28.8	53.9 ± 31.0	0.06 ± 0.32	0.11 ± 0.50	0.12 ± 0.47	0.11 ± 0.51
**RV**	57.6 ± 30.9	51.8 ± 14.4	51.5 ± 14.2	51.4 ± 14.71	0.63 ± 3.20	0.14 ± 0.96	0.13 ± 0.87	0.13 ± 0.87

Table A.6. Double extended source case - Mean and standard deviation of Distance of Localisation Error (DLE) and Intersection over Union (IoU) for different SNR values.

	DLE [mm]				Iou [%]			
	30 dB	20 dB	10 dB	0 dB	30 dB	20 dB	10 dB	0 dB
**Deep-MEG**	30.3 ± 16.3	30.7 ± 16.5	30.9 ± 15.3	31.8 ± 15.3	9.98 ± 10.1	9.89 ± 10.3	9.82 ± 9.71	8.84 ± 10.1
**LCMV**	44.1 ± 15.2	48.0 ± 11.9	48.6 ± 10.5	48.8 ± 10.2	1.13 ± 2.93	0.24 ± 1.28	0.05 ± 0.54	0.05 ± 0.55
**eLORETA**	20.8 ± 15.1	30.6 ± 22.6	58.5 ± 20.7	60.6 ± 19.3	14.5 ± 7.96	9.44 ± 9.50	0.62 ± 2.76	0.29 ± 1.59
**MNE**	61.3 ± 23.6	53.1 ± 22.6	48.2 ± 21.4	56.5 ± 23.2	0.12 ± 0.60	0.15 ± 0.68	0.16 ± 0.59	0.13 ± 0.64
**RV**	49.4 ± 10.6	48.7 ± 10.3	48.2 ± 10.1	48.1 ± 10.1	0.02 ± 0.23	0.07 ± 0.73	0.07 ± 0.74	0.08 ± 0.74
